# Cigarette Waste in Popular Beaches in Thailand: High Densities that Demand Environmental Action

**DOI:** 10.3390/ijerph15040630

**Published:** 2018-03-29

**Authors:** Nipapun Kungskulniti, Naowarut Charoenca, Stephen L. Hamann, Siriwan Pitayarangsarit, Jeremiah Mock

**Affiliations:** 1 Faculty of Public Health, Mahidol University, Bangkok 10400, Thailand; nipapun.kun@mahidol.ac.th; 2 Center of Excellence on Environmental Health and Toxicology, Bangkok 10400, Thailand; 3 Tobacco Control Research and Knowledge Management Center, Bangkok 10400, Thailand; slhamann@gmail.com (S.L.H.); pitayarangsarit@gmail.com (S.P.); 4 Insight Analysis Group, Corte Madera, CA 94925, USA; jeremiah.mock@gmail.com

**Keywords:** tobacco product waste, cigarettes, butts, tobacco, water pollution, marine environment, beach, Sustainable Development Goals (SDGs), Thailand, low- and middle-income countries, (LMICs), Association of Southeast Asian Nations (ASEAN), policymaking

## Abstract

Thailand, like all nations, has a responsibility to initiate environmental actions to preserve marine environments. Low- and middle-income countries face difficulties implementing feasible strategies to fulfill this ambitious goal. To contribute to the revitalization of Thailand’s marine ecosystems, we investigated the level of tobacco product waste (TPW) on Thailand’s public beaches. We conducted a cross-sectional observational survey at two popular public beaches. Research staff collected cigarette butts over two eight-hour days walking over a one-kilometer stretch of beach. We also compiled and analyzed data on butts collected from sieved sand at 11 popular beaches throughout Thailand’s coast, with 10 samples of sieved sand collected per beach. Our survey at two beaches yielded 3067 butts in lounge areas, resulting in a mean butt density of 0.44/m^2^. At the 11 beaches, sieved sand samples yielded butt densities ranging from 0.25 to 13.3/m^2^, with a mean butt density of 2.26/m^2^ (SD = 3.78). These densities show that TPW has become a serious problem along Thailand’s coastline. Our findings are comparable with those in other countries. We report on government and civil society initiatives in Thailand that are beginning to address marine TPW. The solution will only happen when responsible parties, especially and primarily tobacco companies, undertake actions to eliminate TPW.

## 1. Introduction

The marine waters of Southeast Asia are the lifeblood of the region’s existence, as throughout the rest of the world. Now more than ever, the world’s seas and oceans are in urgent need of protection and revitalization because they are irreplaceable primary habitats. Seas and oceans have sustained life on Earth for much longer than the existence of modern humans. For over a century, we humans have been causing unprecedented changes in conditions in marine habitats and these changes will largely determine the survival of marine plants and animals [1], as well as the survival of the human species.

The United Nations Sustainable Development Goals (SDGs) and the 2017 UN Oceans Conference in New York have led to renewed commitments to clean up marine environments and countries are now considering taking actions [2,3]. The SDGs have been adopted by over 190 nations in response to the need to address multiple determinants of national development through a coordinated effort focusing on seventeen goals. Many of the goals deal with environmental issues. Goal 14 in particular specifies measures for protection of the oceans and their revitalization [2].

Many processes like ocean acidification and dispersal of plastic pollution are destroying marine habitats and threatening marine species. Challenges remain in understanding how these processes might be mitigated. However, it is clear that all stressors of marine ecosystems must be reduced to have any chance of maintaining a sustainable marine ecological balance [4].

Developing interventions for environmental improvement requires substantial evidence about the problems and relevant information for policy actions. This paper presents the analysis of data about one aspect of pollution at the interface between land, humans and sea, tobacco product waste (TPW) in beaches. We present policy options to deal with TPW that have emerged from evidence.

In Thailand, marine plastic pollution, including non-biodegradable cigarette butts that persist in the environment, has gained attention recently [5]. A convergence of factors presents an opportunity for more action to address this problem through public policy, including Thailand’s 2017 Tobacco Product Control Act [6]. However, policymakers have given little attention to TPW. The problem of plastic pollution from land-based sources has been neglected due to a lack of public awareness [7]. Another impeding factor is the concealment of the problem by manufacturers of tobacco products who aggressively avoid being held responsible for TPW [8]. 

Recent studies outside of Thailand have shown detrimental impacts of tobacco product pollution and they have suggested possible approaches to address this problem. Novotny et al. conducted toxicity studies showing the types and levels of toxic constituents in discarded TPW, mostly cigarette filters [9,10]. They have also discussed some possible policy options to address these dangers [11], and they have proposed an extended producer responsibility framework to eliminate the problem [12]. However, given that their research focused on conditions in high-income countries, it is important to investigate TPW conditions in low- and middle-income countries (LMICs) like Thailand where public beach use is one of the most important leisure activities for locals and international tourists, and where hundreds of millions of people obtain their basic sources of protein.

### Cigarette Pollution in Beaches Causes Multiple Harmful Impacts

Cigarette smokers pollute beaches through smoke as air pollution and through solid waste from discarded cigarettes (i.e., tobacco, paper, and filters), spent cellulose acetate (plastic) butts, plastic and paper packaging, matches, and lighters. On Thailand’s beaches, as throughout much of the world, cigarette smoke becomes point source air pollution when it drifts through the air in lounge areas, particularly when roofs and structures such as beach umbrellas limit the dissipation and dilution of smoke. In Thailand, conditions on public beaches are considerably different from those at secluded private beaches. At public beaches, 10–20 m back from the water’s edge, one finds thousands of lounge chairs for rent where, under a patchwork cover of beach umbrellas, beachgoers relax and enjoy food and drinks for many hours, taking an occasional swim or playing with children at the water’s edge. Elsewhere, we have reported on cigarette smoke air pollution levels on two of Thailand’s most popular public beaches, which were also sampled in this study [13]. 

Solid TPW pollutes beaches and surrounding areas, often finding its way into marine habitats as a result of smokers littering directly into the water or through drainage from gutters, movement down adjacent waterways, and coastal drainage off the beaches resulting from tidal movements and heavy rains. One study in Europe found that of the various categories of debris that made up marine plastic pollution, TPW was the highest percentage category of items (13% or more) [14]. A large volume of marine TPW comes from rivers flowing into the seas and oceans, as well as from coastal pollution [15]. TPW is the most common item of plastic pollution found in beach clean ups, even greater in number than plastic bottles [16]. 

In Thailand, as throughout coastal Asia, plastic pollution is a serious problem. In 2015, Thailand was ranked the sixth worst plastic polluting country in the world in terms of volume of inadequately managed plastic waste, behind China, Indonesia, the Philippines, Vietnam, and Sri Lanka and just before Egypt, Malaysia, Nigeria, and Bangladesh [17]. Unfortunately, in Thailand most of the public and many international tourists have shown relatively little concern about marine pollution. Until recently, there has been even less concern about the environmental health impacts of TPW. Thailand’s coastal areas are littered with all manner of solid waste, including plastic bottles, plastic bags and plastic cigarette butts. Many smokers use the beaches as though they were an ashtray.

Small particles of plastics, known as micro-plastics, are a significant threat to marine ecosystems. Micro-plastics will not be reduced until TPW is eliminated. Presently, it is estimated that worldwide five trillion micro-plastic particles circulate throughout the oceans, resulting in both water and food contamination and toxicity [18]. Micro-plastics result from the breakdown of larger plastic items, including cigarette filters, which undergo a reduction in size because of photo-degradation and other chemical breakdown processes. Micro-plastics can harm marine plants and animals and even produce harm in humans through chemicals and bacteria that cling to them and move through the food chain [18,19]. Additionally, when TPW enters seas and oceans, toxic and carcinogenic substances contained in tobacco and cigarette butt filters are dispersed into marine waters. These substances have been shown to kill fish and other marine organisms [9].

As with low-dose effects from tobacco smoke, toxins from water-borne TPW are especially dangerous for infants and children. Tobacco and cigarette butts contain ammonia, formaldehyde, benzene, butane, acrylonitrile, toluene, and alkaloid nicotine [9]. Biological monitoring shows that toxins from cigarette butts can have substantial acute effects in children at much lower levels than in adults [20]. For example, a toxic dose of nicotine for an adult is 4–8 mg, while 1–2 mg may be toxic for young children [21]. Heavy metal pollution such as lead is known to have more pronounced acute effects and cause long-term neurological damage in children. Lead, cadmium, and other toxic heavy metals are leached from cigarettes and butts [22] and pollute coastal marine environments [23]. Recent attention has also been given to pollutants classified as endocrine-disrupting chemicals (EDCs) that can have cross-generational effects [24]. Thus, exposures to EDCs, fine particles, and polycyclic aromatic hydrocarbons from air and water pollution from TPW must be reduced, especially for children [24,25]. Since it is clear that infant health can be improved by limiting exposure to tobacco constituents, controlling TPW seems to be doubly worthwhile for both individuals and marine environments [26].

The quality of water in Southeast Asia is declining at an alarming rate. Fresh water and seawater are becoming polluted with a variety of waste products resulting from consumerism, transportation, and housing [27,28]. Some people think of beaches and coastlines simply as leisure areas for snorkeling, scuba diving, parasailing, sport fishing, windsurfing, water sports, clam digging, running, and swimming. Human leisure activity is actually only a very small part of the significance of beaches. Coastal reef fisheries in Southeast Asia are an important source of animal protein, generating over 2.4 billion pounds of seafood per year [29].

People in other regions have also become concerned with coastal marine pollution including TPW. In Spain, urban beach areas and destination beaches were found to have high densities of TPW. The density of litter items was higher at beaches where river outlets were located nearby [30,31]. An Italian study also identified urban areas as the main drivers of marine litter [32]. A multi-country study of coastal litter in the European Union in 2016 using OSPAR guidelines for monitoring marine litter found large quantities of TPW among 50 categories of debris collected [14].

Studies in the Americas show that beachgoers in Brazil, Mexico, and the U.S. value cigarette butt-free beaches [33,34,35]. In Brazil, undeveloped beaches were found to have high densities of marine debris from rivers draining from urban areas [33]. In the Yucatan, cigarette butt litter was found to be a major aesthetic concern for beachgoers [34]. A study in the U.S. focused on how habits, attitudes, awareness of the environmental consequences, and place attachment influence littering behavior [35].

In Africa, the problem of TPW reflects the problems of plastic waste management in many middle-income countries like Thailand. In Morocco, an investigation of polymer wastes found a range of densities of debris with the highest densities in urban areas, then in villages, and <10% on remote/rural beaches. TPW followed this same pattern, with cigarette butts constituting 32% of polymer waste items on urban beaches [36]. A study in South Africa found debris of around 2–25 mm to be most densely concentrated close to urban-industrial centers. This study showed a need to assess local conditions when developing mitigation plans [37].

Conditions at beaches reflect the conditions of marine life in general in a region. Worldwide, the decline in marine biodiversity has become evident with the decline of numerous fresh and seawater species. One report showed that since 1970, land and water species have declined by 58%, with 81% of freshwater species declining in that same period [1]. Coral reefs have been hard hit by human activity that causes pollution, warming water temperatures, and ocean acidification. Recent evidence has shown that these factors are killing coral, including along Thailand’s coast [38,39]. Earth’s current global warming trajectory puts half the world’s corals at risk of dying by 2050 and 90 percent at risk by 2100. Presently, about 275 million people worldwide depend directly on coral reefs for their livelihoods and sustenance [40]. Given the urgent problems of threatened marine ecology throughout Thailand and other coastal areas in Asia, the aim of this study was to determine the levels of TPW polluting some of Thailand’s most popular beaches, and discuss actions that should be undertaken to mitigate the problem of marine TPW pollution.

## 2. Methods 

We used two methods to assess the degree of beach pollution from TPW in Thailand’s beaches. First, we conducted a cross-sectional observational survey of two of Thailand’s most popular public beaches within driving distance of Bangkok. First, we documented the availability and use of tobacco products near the beaches. Then, to determine the density of cigarette butts present on the beaches, we collected samples in beach areas where people congregate—in lounging areas ten or more meters back from the water’s edge and along the open beach. We conducted our surveys on two consecutive weekends in July, one weekend at each beach, at a time of the year after the school vacation season when beach use was not at its peak. On each beach, on two consecutive days over an eight-hour period each day, research staff collected cigarette butts and packaging materials visible on the surface of the sand over a one-kilometer stretch of beach area.

**Figure 1:**
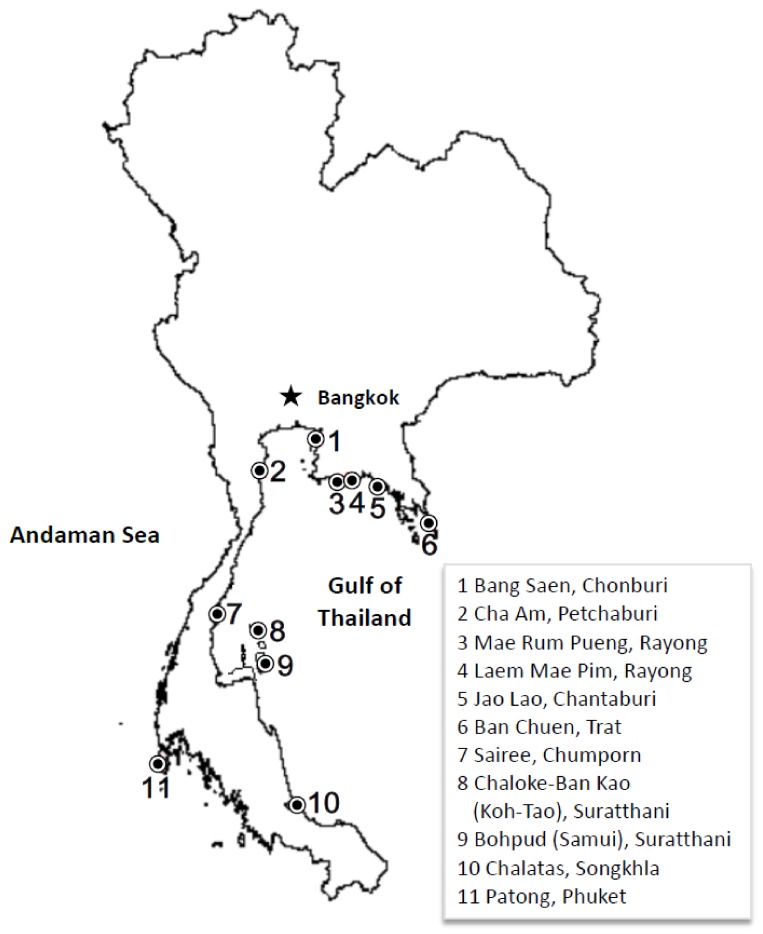
Map of Thailand showing study beaches.

Second, to study conditions at other popular beaches in Thailand and to compare data collection methods, we compiled and analyzed data from sieved sand surveys conducted in 2017 by the Department of Marine and Coastal Resources (DMCR) [41,42,43,44,45,46,47]. DMCR staff surveyed 11 popular beaches throughout Thailand’s coastline, including the two beaches of our initial survey. Ten of the beaches are located along the Gulf of Thailand. Patong beach in Phuket is on the Andaman Sea (see Figure 1). At each of the 11 beaches, DMCR staff collected ten samples of sand within a one-thousand-meter beachfront area 20 m back from the water’s edge. Samples were collected in plots with a surface area of 1 m^2^ and 10 cm of depth. Staff sieved the sand from each sample plot using a ten-mesh sieving screen, recorded the number of butts, and calculated the average butt density per square meter of beach. The possible total number of cigarette butts at each beach was estimated based on the estimated butt density multiplied by the total beach area. We calculated densities based on a standard area of 1000 m × 20 m.

## 3. Results

### 3.1. Cigarette Butt Density Estimated from Surface Collection at Two Public Beaches

At Bang Saen and Cha Am, we found dozens of shops near the beaches selling cigarettes. At Bang Saen, drink carts on the beach were also selling cigarettes. Some carts even displayed cigarette packages in violation of Thailand’s ban of point-of-sale displays of cigarette packs for licensed retailers. At Cha Am carts were not allowed on beachfront areas, but cigarettes were sold in shops across the road from the beach. About half of the shops also displayed cigarette packages in violation of the ban on point-of-sale displays.

We found that smoking was common in the beach lounge areas and along the beach. In our study reported elsewhere on secondhand smoke exposure at these two beaches [13], the density of smokers in sampled zones in an area of about 20 m × 20 m with 50–100 beachgoers was up to four smokers. This results in an estimate of 50 smokers along a 250-meter-long beachfront area.

Our survey produced an estimated density of cigarette butts on the surface of the sand in the beach lounge areas where beachgoers and venders spend most of their time of 0.36/m^2^ at Bang Saen and 0.52/m^2^ at Cha Am (Table 1). Overall, our research staff collected 3067 butts in lounge areas at the two beaches, giving an average butt density of 0.44/m^2^. We found very few roll-your-own butts on either beach. We also found minimal cigarette packaging.

Along the beach, the densities were substantially lower (0.07/m^2^ at Bang Saen and 0.07/m^2^ at Cha Am). Here as well, we found very few roll-your-own butts or cigarette packaging. We found that over the whole area (lounge area and along the beach) the butt densities were 0.12/m^2^ at Bang Saen and 0.14/m^2^ at Cha Am.

### 3.2. Cigarette Butt Density Estimated from Sieved Sand Samples at 11 Beaches

At all 11 beaches sampled using the sieved sampling method, the densities of cigarette butts were high along the beach on the surface and buried down to a depth of 10 cm (see Table 2). Densities of accumulated butts in the sand ranged from 0.25–13.3 per m^2^. Banchuen beach in Trat Province had an unusually high density of butts. The beach at Jao Lao, Chantaburi had the lowest density, nearly half that of the beach with the next lowest density. The mean density of butts collected at the 11 beaches was 2.26/m^2^ (SD = 3.78).

A comparison of the results generated using the two butt collection methods at Bang Saen and Cha Am (in Table 1 and Table 2, Beaches 1 and 2 respectively) showed that the method of surface collection along the beach generated butt densities that were just 11% of the densities estimated using the collection method of sieving samples of sand at a depth of 10 cm. This result shows that 89% of butts accumulated on these two beaches were buried in the sand out of sight.

## 4. Discussion

Our study confirms that at popular beaches throughout Thailand, TPW pollution in beach sand is a serious problem, particularly in beach lounge areas. Our study also shows that to produce an estimate of the accumulated number and density of cigarette butts in a beach, the method of sieving sand in plots to a depth of at least 10 cm is superior to collecting butts visible on the surface. Collecting deeper samples revealed that large quantities of butts had accumulated below the surface over time. Moreover, our findings suggest that a sizable fraction of butts deposited in beach lounge areas were being pulled by the movement of seawater at high tides out into the open beach toward the sea.

Our findings describe the conditions of TPW at recreational beach areas in Thailand that are economically dependent on tourism from local vacationers and international tourists. The butt densities reported here from off-peak periods in June and October likely do not represent the maximum densities that would occur during the height of the Thai vacation season (March–May) or during the peak season of international tourism (November–February).

Our findings are similar to those reported in other studies. One study in Brazil found 9.1 beach debris items per meter of shoreline at one beach location [33]. Various studies of beach debris in Europe have found 9–36% of beach debris are cigarette butts. Our average of 2.26 butts/meter falls between the 9–36% of 9.1 debris items per meter (between 0.82 and 3.28 butts) and at the higher end of that range. Findings from countries outside of Europe give much higher total counts of all types of debris but results from Oman, Mexico, Israel, and Indonesia give overall debris counts in a wide range from 0.4–15.2 debris items per meter of shoreline. Because typically about 9–36% of debris counts are butts, our finding of an average of 2.26 butts/meter is also consistent with these findings.

At the two beaches where we collected cigarette butts from the surface of the sand, we found that Bang Saen had fewer butts in the beach lounge area than at Cha Am, likely due to the posting of some “no smoking” signs in the beach lounge area where beachgoers rented chairs for lounging and where maintenance personnel raked and cleaned the sand. The number of roll-your-own butts was higher at Cha Am, most likely because the local population frequented Cha Am more than Bang Saen.

Empty cigarette packages were surprisingly scarce at both beaches, probably because maintenance personnel cleaned both beaches to remove the most visible trash left on the beach. Although we lack information about the type and frequency of sand cleaning at the 11 beaches surveyed, we know that most of the beaches catered to international tourists and maintenance personnel at these beaches removed cigarette butts and other waste. None of the beaches had mechanical equipment to clean beach sand. Even though some of these beaches had been raked manually, substantial numbers of cigarette butts had accumulated. At Banchuen, Trat, the high density of butts was likely due to the abundance of small open cabanas that allowed resort residents to throw their cigarette butts into the sand below. At Jao Lao, Chantaburi, the low density of butts was likely due to the efforts of local residents and resort operators to reduce the volume of cigarette butts in their beach. The variability in butt density per sample could be due to the proximity to beach resorts or hotels, and the degree to which care of the beaches included raking the beach sand to remove cigarette butts. Another possible factor, one identified in many studies worldwide, was the proximity of the beaches to water outlets from street drainage, streams, and rivers. Many cigarette butts are deposited on beaches because they are carried from upstream sources out to the sea and deposited by tidal flows on beaches. In some beaches, fewer butts may have been due to lighter beach use and better care of beaches critical to tourism.

Unfortunately, our study cannot confirm conclusions drawn in other studies regarding cigarette butt pollution around urban centers and near outlets from drainages, streams, and rivers emptying into the sea [32]. The beaches we investigated were vacation beaches near resorts and hotels, but not near urban centers. In our study, we believe the specific local circumstances—how the beach-going public used the beaches, the convenient availability of cigarettes, and the services available for beach lounging—had an important effect in producing high butt densities.

### What Should Be Done?

Our finding of large quantities of cigarette butts in Thailand’s beaches reveals a general lack of public awareness and concern about the hazardous pollution caused by TPW. This revelation is not surprising considering the overall high level of plastic waste at Thailand’s popular beaches. It is also not surprising given that about 90% of TPW was buried below the surface of the sand, out of sight and therefore out of mind. Additionally, concerns about outlets discharging TPW into the sea have not been addressed thoroughly at the locations we studied. In the past, we have investigated various types of stream, river, and sewage discharges in Thai coastal waters and we have found that wastewater discharges are loaded with many type of pollutants, including TPW [48]. TPW from tributary flows and discharges need to be eliminated, especially in newly urbanizing areas along Thailand’s coast.

In our review of the literature, we found that there is no standardized international protocol for estimating accumulated TPW in beach sand. Our study has demonstrated that the method of collecting samples in 1 m^2^ plots at a depth of 10 cm better characterizes TPW on Thailand’s beaches and should be considered as a useful method in future studies. A rigorous standardized protocol is needed to allow for international comparisons.

In many countries, activists have used beach waste counts as evidence to highlight problems of rapidly increasing marine pollution caused by waste, especially plastic waste of all sizes, including TPW [49]. However, conditions vary so widely in terms of pollution sources from remote to highly urbanized and urbanizing locations that it is impossible to generalize when characterizing the sources of marine debris. What is clear is that TPW is widespread globally and that it creates environmental impacts due to the product’s design, materials used, and human behavior. Physical features of land and water pollution contribute to the distribution of TPW, but they alone neither determine the origin of the waste nor establish the basis of responsibility that should be borne for such avoidable pollution.

We plan to continue examining the sources and causes of TPW with greater precision. To summarize, our findings show that:

- Thailand’s popular beaches are heavily polluted with cigarette butts and other TPW.
- TPW is deposited throughout beaches, but especially in higher densities where beachgoers congregate in large numbers in sheltered beach lounge areas.
- TPW is gradually becoming recognized as a major environmental pollutant in Thailand, as evidenced by the DMCR’s efforts to collect samples of butts at popular beaches.
- The disposable mindset and careless habits that result in beaches being polluted with TPW will have to be addressed through regulatory, cultural and behavioral strategies.

While environmental scientists and tobacco control researchers have recognized the harms of TPW pollution, policymakers have only recently started to act. In Thailand, policymaking has often been catalyzed by voluntary efforts undertaken by civil society groups. Policy measures have included self-enforced laws designating smoke-free zones, rules on littering, provision of mobile or stationary cigarette waste receptacles, and public education. These measures have administrative and enforcement costs to the government and for the parties that implement them. So, elimination of TPW will require a sustained administrative and financial commitment.

Policymakers often choose “product stewardship” approaches that put most of the responsibility for reducing TPW on the consumer and the general public. Companies that produce products that result in post-consumption waste readily support this approach because they can avoid taking responsibility for waste that results from using their products. However, we believe that producers of TPW, particularly tobacco companies, should pay for the safe and complete disposal of the hazardous waste their products create, as is required of many other companies that produce products with hazardous components and ingredients such as cell phones, paint, and pesticides. This practice is known as “extended producer responsibility” and it has been implemented in some countries as a way to pay the costs of removing TPW [50,51].

Examination of past efforts to force tobacco companies to accept responsibility for post-consumption waste shows that these companies aggressively fight such requirements. For example, although the City and County of San Francisco, California implemented a cigarette litter abatement fee to pay for cleanup costs, tobacco companies challenged this approach, resulting in a referendum to stop the adoption of such fees in other localities [52]. Nevertheless, approaches like added TPW mitigation fees on tobacco products could be adopted in Thailand, along with smoke-free laws that further restrict where smoking is allowed. Mitigation fees are a means to recoup the costs of administering and implementing TPW cleanup programs to make the producers pay for cleanup, while discouraging smoking by making manufacturers pass down their true costs to the public and the environment.

Policy tools for dealing with TPW can be used in Thailand with a combination of voluntary and regulatory measures to forward the agenda of preventing TPW pollution. The development of these measures would require the exclusion of the tobacco industry from policymaking to prevent a conflict of interest. The needed innovations should shift the environmental and disposal costs of TPW to the producers, not the consumers. Policymakers should also use innovative means to eliminate the use of plastic filters because these plastic components of TPW provide no health benefit whatsoever to cigarette smokers [53].

In Thailand, the 2017 Tobacco Product Control Act states that the Ministry of Public Health can propose new regulations such as a ban on tobacco use on beaches. The Ministry of Natural Resources and Environment has already undertaken actions to ban smoking on 24 heavily used public beaches throughout Thailand to prevent marine pollution from TPW. It is likely that this ban will be extended to all beaches in Thailand [54]. Additionally, grassroots groups in Thailand like Trash Heroes, which started in 2013 and has expanded to other countries in Southeast Asia, are organizing beach cleanups, offering education programs, and advocating for stronger laws and regulations on marine pollution. In the Association of Southeast Asian Nations (ASEAN) region, Trash Heroes recently presented their work at the 2017 ASEAN Conference on Reducing Marine Debris held in Phuket, Thailand. At this meeting, for the first time, ASEAN countries pledged to take actions to reduce single-use plastics, particularly TPW [55].

The convergence of public and civil society actions makes the possibility of an improved marine environment real in Thailand. Adopting feasible and innovative approaches creates awareness in the public and among environmental institutions that we believe will result in commitments to revitalize the seas and oceans.

## 5. Conclusions

TPW is a significant factor contributing to major changes in marine ecosystems. Thailand, like all other coastal LMICs, faces a massive challenge of cleaning up beaches and protecting marine habitats and species. Much of the TPW pollution in beaches along Thailand’s coastline eventually ends up in the seas and oceans. Accordingly, people and policymakers must become innovative to mitigate the mounting problems of marine ecological degradation caused by TPW.

The SDGs highlight how overcoming ocean pollution is an integral part of achieving global sustainability. Thus, every nation should assess their TPW problems and define the role they can play in caring for the seas and oceans. Legal and regulatory authorities have developed avenues and tools for addressing this problem. Now that awareness about the hazards of TPW is increasing, civil society groups should assume a greater role in monitoring TPW and they should undertake actions to reduce marine debris. We urge tobacco companies, policymakers and all citizens to take responsibility for eliminating TPW in marine environments to protect our world’s threatened seas and oceans.

## Figures and Tables

**Table 1:** Density of cigarette butts on the surface of sand at two popular public beaches in Thailand.

Type of Tobacco Waste	Number (Density)		
	1. Bang Saen	2. Cha Am	Totals
*Beach lounge area*			
Cigarette butts	1436 (0.36/m^2^)	2171 (0.52/m^2^)	3607 (0.44/m^2^)
Roll-your-own butts	21	98	119
Empty packages	6	4	10
*Along the beach*			
Cigarette butts	1345 (0.07/m^2^)	1340 (0.07/m^2^)	2685 (0.07/m^2^)
Roll-your-own butts	8	57	65
Empty packages	8	8	16
*Total counts*			
Cigarette butts	2781 (0.12/m^2^)	3511 (0.14/m^2^)	6292 (0.13/m^2^)
Roll-your-own butts	29	155	184
Empty packages	14	12	26

**Table 2:** Cigarette butt density estimated from sieved sand at 11 beaches along Thailand’s coastline, 2017.

Name of Beach	Butts/m^2^	Total Beach Area (m^2^)	Number of Cigarette Butts
1. Bang Saen, Chonburi	0.62	171,742	106,480
2. Cha Am, Petchaburi	0.62	341,000	211,420
3. Mae Rum Pueng, Rayong	0.98	650,492	637,482
4. Laem Mae Pim, Rayong	2.30	48,900	112,470
5. Jao Lao, Chantaburi	0.25	34,976	8744
6. Banchuen, Trat	13.30	38,400	510,720
7. Sairee, Chumporn	1.05	49,893	52,378
8. Bohpud (Samui), Suratthani	0.99	25,938	25,679
9. Chaloke-Ban Kao (Koh-Tao), Suratthani	0.45	6829	3073
10. Chalatas, Songkhla	3.56	49,719	177,000
11. Patong, Phuket	0.76	132,895	101,000
Average butt density overal 11 beaches	2.26		

Source: Department of Marine and Coastal Resources.
